# High angular diffusion tensor imaging estimation from minimal evenly distributed diffusion gradient directions

**DOI:** 10.3389/fradi.2023.1238566

**Published:** 2023-09-11

**Authors:** Zihao Tang, Sheng Chen, Arkiev D’Souza, Dongnan Liu, Fernando Calamante, Michael Barnett, Weidong Cai, Chenyu Wang, Mariano Cabezas

**Affiliations:** ^1^ School of Computer Science, The University of Sydney, Sydney, NSW, Australia; ^2^ Brain and Mind Centre, University of Sydney, Sydney, NSW, Australia; ^3^ Faculty of Medicine and Health, The University of Sydney, Sydney, NSW, Australia; ^4^ School of Biomedical Engineering, The University of Sydney, Sydney, NSW, Australia; ^5^ Sydney Imaging, The University of Sydney, Sydney, NSW, Australia; ^6^ Sydney Neuroimaging Analysis Centre, Sydney, NSW, Australia

**Keywords:** deep learning, MRI, DWI, DTI, high angular resolution, fractional anisotropy

## Abstract

Diffusion-weighted Imaging (DWI) is a non-invasive imaging technique based on Magnetic Resonance Imaging (MRI) principles to measure water diffusivity and reveal details of the underlying brain micro-structure. By fitting a tensor model to quantify the directionality of water diffusion a Diffusion Tensor Image (DTI) can be derived and scalar measures, such as fractional anisotropy (FA), can then be estimated from the DTI to summarise quantitative microstructural information for clinical studies. In particular, FA has been shown to be a useful research metric to identify tissue abnormalities in neurological disease (e.g. decreased anisotropy as a proxy for tissue damage). However, time constraints in clinical practice lead to low angular resolution diffusion imaging (LARDI) acquisitions that can cause inaccurate FA value estimates when compared to those generated from high angular resolution diffusion imaging (HARDI) acquisitions. In this work, we propose High Angular DTI Estimation Network (HADTI-Net) to estimate an enhanced DTI model from LARDI with a set of minimal and evenly distributed diffusion gradient directions. Extensive experiments have been conducted to show the reliability and generalisation of HADTI-Net to generate high angular DTI estimation from any minimal evenly distributed diffusion gradient directions and to explore the feasibility of applying a data-driven method for this task. The code repository of this work and other related works can be found at https://mri-synthesis.github.io/.

## 1. Introduction

Magnetic Resonance Imaging (MRI) is a non-invasive imaging technique based on the principles of Nuclear Magnetic Resonance to reconstruct detailed images of the internal structure of the human body. Since its inception, several MRI modalities have been proposed to probe specific information about different properties of multiple tissues and organs of interest. On one hand, conventional structural MRI sequences, such as T1- and T2-weighted images, can provide valuable morphological details of the brain and pathological conditions. On the other hand, Diffusion Weighted Imaging (DWI) can measure the water diffusivity within tissues and reveal their microstructure and integrity (1). DWI is extensively used in clinical neuroscience research because it is particularly sensitive to the diffusivity of water molecules in brain tissues and it can help to reconstruct fibre bundles and estimate brain connectivity. DWI volumes are acquired by applying different diffusion-sensitising gradients (b-vectors) with different magnetic field strengths (b-values) to precisely characterise the movement of water molecules in different directions as a measure of the resulting changes in signal intensity. As a consequence, the number and strength of the gradients used determine the level of detailed characterisation of diffusivity.

Each set of images at different gradient directions can be summarised using a Diffusion Tensor Imaging (DTI) model to uncover microstructural information by describing water’s directionality and its corresponding quantitative anisotropy (2). The diffusion of a particular voxel can be characterised as an ellipsoid that can be mathematically formulated as a symmetric 3×3 tensor matrix. For the purpose of individual and group-wise analysis (3), several diffusion scalar measures can be further derived to better reveal the properties of that tensor. In clinical settings and neuroscience research, fractional anisotropy (FA), axial diffusivity (AD), radial diffusivity (RD), and mean diffusivity (MD) are the most commonly used scalar measures. In particular, FA and MD are metrics that provide information about the degree of diffusion anisotropy and the overall magnitude of diffusion at a voxel, respectively. AD, on the other hand, quantifies the magnitude of diffusion along the direction of fibre tracts (primary eigenvector). Disparities of the diffusion scalar measures between diseased and healthy control populations can be observed in clinical studies conducted on various neurological disorders, including multiple sclerosis (MS), lateral sclerosis (LS), Alzheimer’s disease dementia (ADD), Parkinson’s disease (PD), schizophrenia, epilepsy, and other diseases resulting in brain damage (4, 5). For instance, pathological factors including oedema, demyelination, gliosis, and inflammation (6) usually lead to a decrement of FA in the impacted regions. These studies utilise scalar measures as observation metrics to identify discriminative biomarkers between patients and controls. For example, DTI has been shown to be effective in identifying multiple sclerosis lesions with severe tissue damage and monitoring tissue changes (7). Specifically, the patient group exhibited lower FA and higher MD in the normal-appearing white matter (8).

However, scalar metrics derived from DTI can be unreliable due to clinical imaging constraints. This is usually caused by sub-optimal acquisition protocols, which often lead to a low angular resolution diffusion image (LARDI) (9). By definition, the symmetric matrix of the DTI model at each voxel contains only 6 unique values (Dxx, Dxy, Dxz, Dyy, Dyz, and Dzz for a tensor D with subindices representing one of the Cartesian axes) and a linear equation system to fit it would only require the same amount of variables to obtain a unique solution. Therefore, 6 diffusion gradient directions are the minimal number of directions required to fit a diffusion tensor model. Yet, clinical scans with a limited number of directions have been found to be less reliable in the derived DTI scalar measures (10, 11) which can lead to erroneous observations for clinical studies.

To address the trade-off between acquisition time and high resolution (both spatial and angular), image enhancement techniques have played an important role in generating high-quality images from their corresponding low-quality acquisitions (12). U-Net (13), a de-facto architecture for a large number of deep learning networks, first introduced the skip connections between the encoder-decoder network structure and its variations have achieved state-of-the-art in image processing tasks, including image enhancement. These methods employ strategies including sub-pixel upsampling, residual learning, pixel loss, self-ensemble, and attention mechanisms (14) and have been extensively used in medical imaging applications. For instance, image enhancement has been applied to structural MRIs to show detailed morphological information of the organs of interest (15, 16). As stated, DWI also introduces the need for a high angular resolution acquisition, which can be potentially solved by scanning protocols (higher cost), upgrades of the acquisition hardware (with limitations on cost and resolution enhancement (12)) or via “software” (image enhancement algorithms). On the latter, a recent work (DeepDTI) proposed to address the issue directly on DWI by improving the quality of a simulated acquisition with ideal gradients (17) but none of them has focused on the minimal number of image gradients required to greatly reduce the cost for DTI models while retaining the information from a high-quality acquisition.

To this end, we propose to estimate an enhanced DTI model from a minimal acquisition of 6 evenly distributed diffusion gradient directions, by exploiting the corresponding information from high angular resolution diffusion imaging (HARDI) training data using a High Angular DTI Estimation Network (HADTI-Net). For the rest of the paper, we define HARDI as a high angular resolution of 90 diffusion gradient directions and LARDI as a sampled DWI set of 6 evenly distributed gradient directions from all the available 90 directions. The corresponding estimated DTI models for HARDI and LARDI are defined as HAR-DTI and LAR-DTI, respectively. We investigated the feasibility of HADTI-Net to attain FA measurements from LARDI that are comparable to those obtained through HAR-DTI.

The major contributions of this work are summarised as follows:

∙ To the best of our knowledge, this is the first work to estimate a HAR-DTI model from a minimal number of evenly distributed diffusion gradient directions.
∙ The proposed High Angular DTI Estimation Network (HADTI-Net) is specifically designed to estimate an enhanced DTI model from LARDI combining the structural information of T1 and b0 with the diffusion gradient directions to mitigate FA disparities when compared to HAR-DTI.
∙ Evaluation has been conducted and compared with clinical evidence for various neurological disorders to prove the effectiveness and consistency of the enhanced LAR-DTI to reduce noisy estimates and recover missing clinical information from LARDI.
∙ Extensive evaluations have been conducted to prove the robustness of the proposed HADTI-Net to estimate an improved and consistent DTI model from LARDI with significantly lower FA differences from any set of 6 evenly distributed directions.

## 2. Materials and methods

### 2.1. Dataset and pre-processing

#### 2.1.1. The human connectome project

The Human Connectome Project (HCP) database (18) includes anatomical T1-weighted imaging and diffusion-weighted imaging acquired using a 3T Siemens “Connectom” Skyra scanner. The high-resolution T1-weighted data were acquired with 0.7 mm isotropic resolution, TR/TE=2400/2.14 ms, and $\text{\textbackslash{},flip angle}=8^{\circ }$. The high-resolution diffusion MRI data were acquired with 1.25 mm isotropic resolution, TR/TE=5520/89.5 ms, and $\text{\textbackslash{},flip angle}=78^{\circ }$. The diffusion MRI protocol consists of three diffusion-weighted shells which refer to the b-values of 1000, 2000, and 3000, with each shell consisting of 90 directions. 100 different subjects were selected from the HCP database in our study. Bias correction was applied to the structural T1 images (19). The corrected images were then registered to the standard MNI space (20) using linear (FLIRT) and non-linear (FNIRT) registration tools from the FSL package (21). The preprocessing steps of the raw diffusion images included corrections for motion, susceptibility distortions, gradient non-linearity, and eddy currents (22, 23). FreeSurfer (24) was used to generate the final brain mask for each subject (25).

#### 2.1.2. DWI derivatives

**Figure 1:**
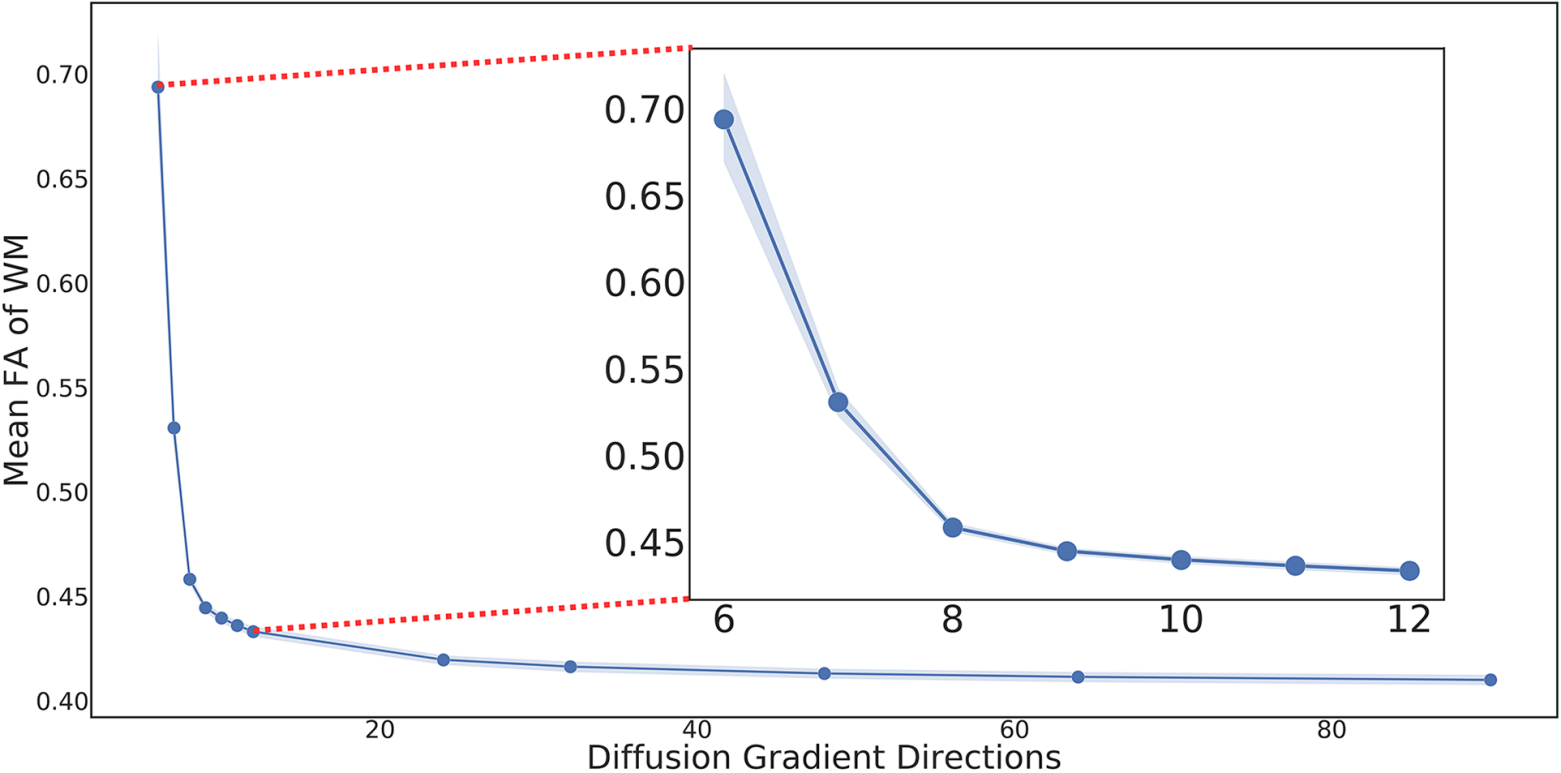
Mean FA of WM in DTIs generated using different numbers of evenly distributed diffusion gradient directions with 95% CI. Due to its nature, the Kennard-Stone algorithm guarantees that the lower number of direction samples are always included in higher ones (e.g., the 6 evenly distributed directions are part of the 12 evenly distributed ones).

To simulate a single shell acquisition, the 90 directions from the b=1000 shell were selected as the ground truth and evenly distributed directions were sampled to simulate the LARDI data. The corresponding diffusion tensor model was fit at each voxel (26) of all the processed LARDIs using DTIFIT from FSL to generate the corresponding DTI volumes with dimensions of 6×145×174×145. Figure 1 illustrates the resulting mean subject FA distribution values for the fitted tensor models with different numbers of sampled directions. The line plot clearly shows how the mean FA values for each sampled DWI set have significant disparities when comparing the minimum and maximum number of directions for the HCP dataset, even though these differences decrease as the number of acquired directions increases. This observation indicates that FA from LAR-DTI is less reliable when measuring potential biomarkers in clinical studies.

Finally, to analyse the performance of HADTI-Net on each individual white matter (WM) tract, TractSeg was used to generate 72 white matter tracts on corresponding HARDI for each testing subject as described in their original paper (27).

#### 2.1.3. Gradient direction sampling

To sample a set of gradient directions from the HARDI acquisition, two variations of the Kennard-Stone (KS) algorithm (28, 29) were used. The original algorithm was developed to sample evenly distributed values from a set by selecting the two first most distant points (given a specific distance function) and additional points are added based on the distance to the current sampled set. In our case, we treat the gradient vectors (bvecs) as points in the hemisphere and use the Euclidean distance for the KS algorithm.

Due to the algorithm’s nature, smaller sets of values are always a subset of larger ones (e.g. the 6 evenly distributed directions are part of the 12 evenly distributed ones). Furthermore, KS is a deterministic algorithm that will always provide the same samples given a specific set of values. As a consequence, there would only be one single set of 6 evenly distributed directions if using the original algorithm for the HCP dataset. To increase the variability of sampled directions and guarantee generalisation to unseen minimal acquisitions, we introduce an extension of the KS algorithm (RandomKS). Specifically, for each subject, a random direction gradient is selected instead of the two furthest apart. Afterwards, the regular KS algorithm is applied to select the furthest value from the current sampled set until 6 directions are selected.

### 2.2. High angular DTI estimation network

**Figure 2:**
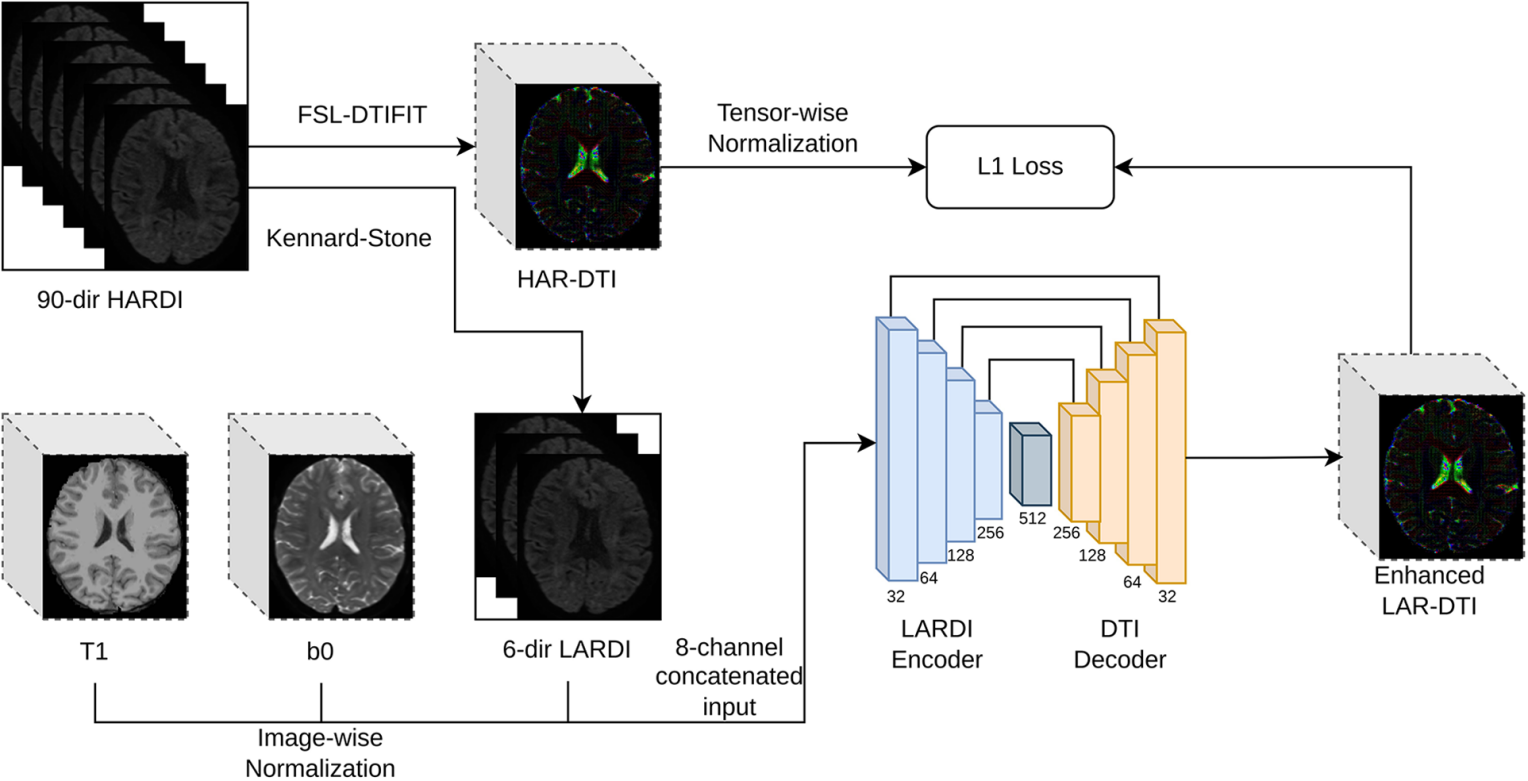
Detailed framework of the proposed HADTI-Net. HADTI-Net takes a concatenated patch of 3D T1-weighted, b0, and diffusion-weighted images with 6 minimal evenly distributed directions to predict an enhanced LAR-DTI.

Common clinical acquisitions rely on a high-quality T1-weighted image for structural information (faster to acquire than DWI due to its shorter scanner time and fewer potential distortion factors) and a single or multiple b0 images (images acquired using the same acquisition parameters as the other diffusion volumes but without any diffusion weighting) as a reference to merge structural information from T1-weighted images and help with the DWI pre-processing. The design of our proposed High Angular DTI Estimation Network (HADTI-Net) is shown in Figure 2. HADTI-Net is based on a 3D U-net (30) and takes an 8-channel concatenated input (T1, b0, and LARDI with 6 evenly distributed directions) and outputs the corresponding enhanced LAR-DTI. Image-wise z-score normalisation was applied to the T1-weighted, b0, and each diffusion-weighted image independently before concatenation. HADTI-Net consists of a LARDI encoder and a DTI decoder. LARDI encoder has 4 convolution blocks and each convolution block consists of two consecutive 3D convolutions with stride and dilation set to 1 and 2, respectively. The filter numbers for the 3D convolution layers in the convolution blocks are 32, 64, 128, and 256, respectively. Similar to the LARDI encoder, the DTI decoder has 4 deconvolution blocks where each block consists of a 3D transposed convolution and followed by a 3D convolution layer which concatenates the residuals from corresponding convolution blocks in DTI encoder as the input. A bottleneck 3D convolution layer with 512 filters is integrated to bridge the LARDI encoder and DTI decoder. The output channels of the LARDI encoder and DTI decoder are provided in Figure 2. Similarly, each tensor coefficient was normalised separately using their z-score and compared to the enhanced LAR-DTI prediction. Neurological disease research has largely focused on the study of white matter tract integrity, thus HADTI-Net was supervised with the L1 loss only in the WM region during the training phase. The final DTI prediction was de-normalised using the mean and standard deviation from the training set.

### 2.3. Implementation details

The 100 preprocessed HCP subjects were split into a training and testing set with a commonly used 80–20% ratio, respectively. The concatenated input had a total of 8 channels which include a channel for T1, a channel for b0, and 6 channels for minimally evenly distributed diffusion gradient directions. The patch size of the input was set to 64×64×64 with a 32×32×32 overlap. We discarded any patches containing only the background region to reduce the computational cost during the training phase. The HADTI-Net was trained for 100 epochs using Adam with an initial learning rate of 0.0001 and batch size of 8 on a single NVIDIA GTX 1080. For inference, the averages from different overlapping regions between adjacent patches were calculated to reconstruct the final enhanced LAR-DTI volume. The code was implemented on PyTorch (version 1.10.1) and Numpy (version 1.21.2).

### 2.4. Metrics and statistical analysis

To evaluate the differences between two sets of DTI volumes (i.e. LAR-DTI and HAR-DTI), we used the following common similarity metrics: mean absolute error (MAE), structural similarity index (SSIM), and normalised cross-correlation. For each metric, we report the mean and standard deviation values of different regions of interest (depending on the experiment) for all the subjects.

Finally, for each set of values, we performed a normalcy test to determine whether the requirements to run t-tests were met. If the requirement was met, paired t-tests were performed to determine differences between paired values (e.g. the metrics for the same subject for the LAR-DTI and enhanced DTI predictions), while independent t-tests were runned when there was no direct way to pair different measurements (e.g. when shuffling the input images). When normalcy was not met, Wilcoxon rank-sum and the Mann-Whitney U statistical sets were run, respectively. For all the hypothesis testing experiments, the level of significance was set at α=0.01 and Bonferroni correction was applied (α/20) when running multiple comparisons.

### 2.5. Experimental design

To validate the proposed algorithm, we performed 4 different experiments, where each one addressing a different research question. These experiments are briefly described as follows:

∙ *What is the gap between our prediction and HAR-DTI from an image similarity perspective for the whole brain? (Section 3.1)*. To address this question, we compute different common image similarity metrics (as described in the previous section) to determine the original gap between the LAR-DTI using KS sampling and the ground truth HAR-DTI and whether that gap was bridged using our enhanced prediction focusing on the whole brain.
∙ *What is the gap between our prediction and the HAR-DTI metrics on different WM tracts? (Section 3.2)*. For this experiment, we focus exclusively on FA which is the most affected metric when using a minimum number of gradient directions. Furthermore, we use the TractSeg segmentations from corresponding HARDI to determine 72 regions of interest.
∙ *How are the prediction errors relevant in clinical practise when analysing group-wise differences according to relevant literature? (Section 3.3)*. First, we performed a literature review for group-wise FA differences in different tracts for neurological disorders. Afterwards, we compare these differences with the margin error obtained by our method to determine whether these group-wise differences could still be observed with the original LAR-DTI model and our enhanced prediction.
∙ *How important is the sampling of gradient vectors and their order? (Section 3.4)*. We propose three different hypotheses that we statistically test to determine the importance of how the diffusion-weighted images are seen by introducing randomness to different aspects during inference.

## 3. Results and discussion

### 3.1. Diffusion tensor analysis

To evaluate the gap between the DTI estimates from LARDI (both the original LAR-DTI and the enhanced predictions by DeepDTI and HADTI-Net) in the whole brain, we calculated four different DTI-derived scalars commonly used in clinical practice (FA, MD, AD, and RD). The results are summarised separately for WM and GM in Table 1. One of the first important conclusions is the need for a robust set of metrics that evaluate different properties and that are not sensitive to the scale of the intensity values. While MAE is commonly used to estimate the error between the images, its scale might lead to a misinterpretation of the results. For instance, if we focus on AD and RD, it is hard to argue that the HADTI-Net predictions are truly better (even though significant differences were observed). However, looking at SSIM and cross-correlation (metrics that focus on structural information on local and global level respectively), we can clearly see how the AD and RD estimates are subpar for LAR-DTI and we can also observe a large and significant improvement using our proposed model. This is important, as any analysis that focuses on specific regions of the brain or uses structural information based on these images might lead to erroneous conclusions if using LAR-DTI, while using the enhanced predictions would be a better choice due to bridging the gap with HAR-DTI (both SSIM and cross-correlation at bounded with a maximum value of 1).

**Table 1:** Summary of the results for the whole brain region according to the differences on common DTI-derived metrics.

Method	FA	MD	AD	RD
White matter				
MAE ↓				
LAR-DTI	0.2732±0.0810∗	0.0000±0.0000	0.0004±0.0003∗	0.0002±0.0001∗
DeepDTI	0.1349±0.0094∗	0.0000±0.0001	0.0002±0.0002∗	0.0001±0.0000∗
HADTI-Net	0.0595±0.0213	0.0000±0.0000	0.0001±0.0001	0.0000±0.0000
SSIM ↑				
LAR-DTI	0.2652±0.0818∗	0.6069±0.1191	0.2692±0.1024∗	0.2865±0.1078∗
DeepDTI	0.6260±0.0314∗	0.7113±0.1732	0.5024±0.1371∗	0.5932±0.1256∗
HADTI-Net	0.8401±0.0574	0.7337±0.1479	0.7227±0.1038	0.7367±0.1243
Cross-correlation ↑				
LAR-DTI	0.3941±0.1002∗	0.9170±0.0926	0.5511±0.1175∗	0.6016±0.1595∗
DeepDTI	0.7331±0.0633∗	0.9108±0.1432	0.7597±0.1810∗	0.8701±0.1009∗
HADTI-Net	0.8980±0.0671	0.9372±0.0895	0.9019±0.0943	0.9291±0.0772
Grey matter				
MAE ↓				
LAR-DTI	0.3377±0.1022∗	0.0000±0.0000	0.0004±0.0002∗	0.0002±0.0001∗
DeepDTI	0.0597±0.0171∗	0.0001±0.0002	0.0001±0.0002	0.0001±0.0002
HADTI-Net	0.0513±0.0061	0.0000±0.0000	0.0001±0.0000	0.0000±0.0000
SSIM ↑				
LAR-DTI	0.1414±0.0636∗	0.8787±0.0874	0.3989±0.1315∗	0.5761±0.1467∗
DeepDTI	0.6593±0.0592∗	0.8663±0.2063	0.7676±0.1800	0.8455±0.2031
HADTI-Net	0.7295±0.0448	0.8047±0.1246	0.7474±0.1186	0.8003±0.1184
Cross-correlation ↑				
LAR-DTI	0.2794±0.0529∗	0.9669±0.0506	0.6362±0.1302∗	0.7553±0.1435
DeepDTI	0.5268±0.0691∗	0.9341±0.1167	0.8303±0.1266	0.9230±0.1183
HADTI-Net	0.6857±0.0568	0.8614±0.1031	0.8348±0.1080	0.8645±0.1001

${}^{*}$Indicates significantly worse results when compared to HADTI-Net (higher or lower values depending on the metric) with the significance level α=0.01.

A clear observation from the results is the overall improvement of the diffusion scalar metrics for the whole brain (even in the GM region which was not supervised by HARDI-Net). Regarding FA measures, the differences can already be apparently observed exclusively at the MAE values. This is not necessarily surprising as FA images have in general higher intensity values leading to a higher range of error values. Nonetheless, enhanced LAR-DTI by HADTI-Net is capable to obtain the highest SSIM (when compared to MD, AD, and RD) for FA and comparable results for cross-correlation. This is important, as FA is one of the most commonly used metrics for clinical studies as it provides a good proxy to understand anisotropy (even though it is not suitable for regions with crossing fibres). Furthermore, due to its computational complexity (it requires obtaining the eigenvalues of the tensor and it involves a more complex equation than the other three metrics), it is the most prone to numerical errors. Therefore, being able to predict a DTI tensor that is closer to a HARDI acquisition (with 90 directions) with a clinical acquisition is important.

Finally, while improvements are shown for MD, the differences were not found to be significant. In fact, the cross-correlation values for both LAR-DTI and the network predictions are the highest among all the diffusion scalar measures. These results suggest that the MD information is preserved and is not as sensitive to noise in the acquisition. This is to be expected when taking into account the definition of MD and the limitations of the DTI model. MD is related to the magnitude of diffusivity on the three axes of the tensor. As such, it is not much affected by the direction of the tensor (main eigenvector and eigenvalue like AD), or its relationship between axes (like FA would). In conclusion, erroneous LARDI MD measurements may be undetected if the sum of the magnitude of each axis is not changed regardless of the changes of the fitted ellipsoid shapes.

**Figure 3:**
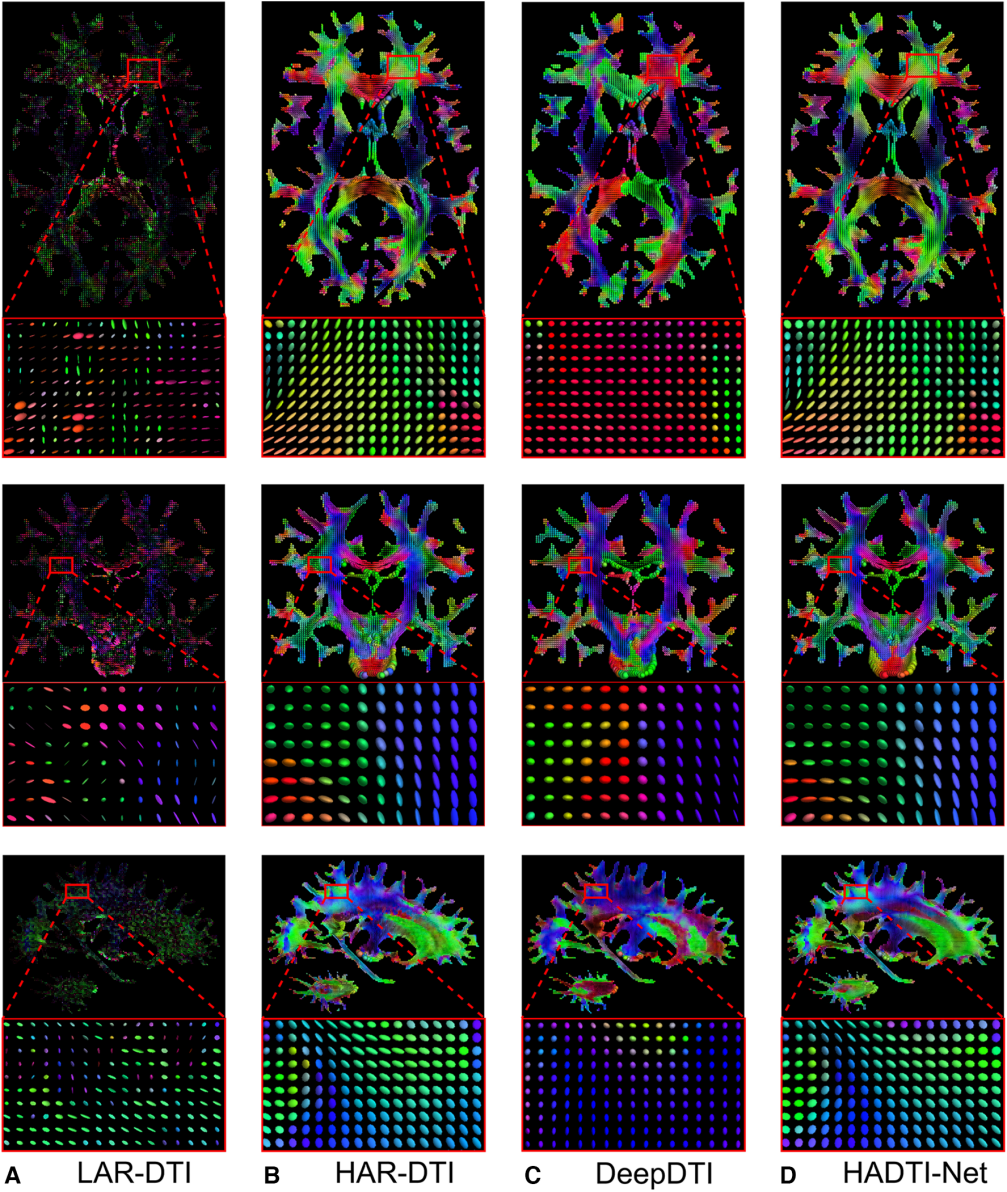
Visualisations of (**A**) LAR-DTI, (**B**) HAR-DTI, enhanced LAR-DTI by (**C**) DeepDTI and (**D**) HADTI-Net using (**A**) as the input for a testing subject. From the top to bottom are the axial, coronal, and sagittal views, each including a zoomed region of interest. The color coding of the diffusion tensor indicates directionality, whereby red, green, and blue represent right-left, anterior-posterior, and inferior-superior directions, respectively.

To better illustrate these results, we visualise the axial, coronal, and sagittal views (and a zoomed-in region) of the diffusion tensors for a testing subject in Figure 3. From the zoomed-in region of interest, we can observe how the network is capable of correcting most of the inaccurate directionality and shape of the diffusion tensors (as discussed with the numerical results). Furthermore, while there might be small differences, it is hard to visually distinguish the gap between the HAR-DTI model and the enhanced prediction. In summary, both the quantitative and qualitative results showed strong disparities between LARDI and HARDI, and HADTI-Net was able to mitigate the differences caused by a minimal set of DWI gradients.

### 3.2. White matter fractional anisotropy analysis on WM tracts

**Figure 4:**
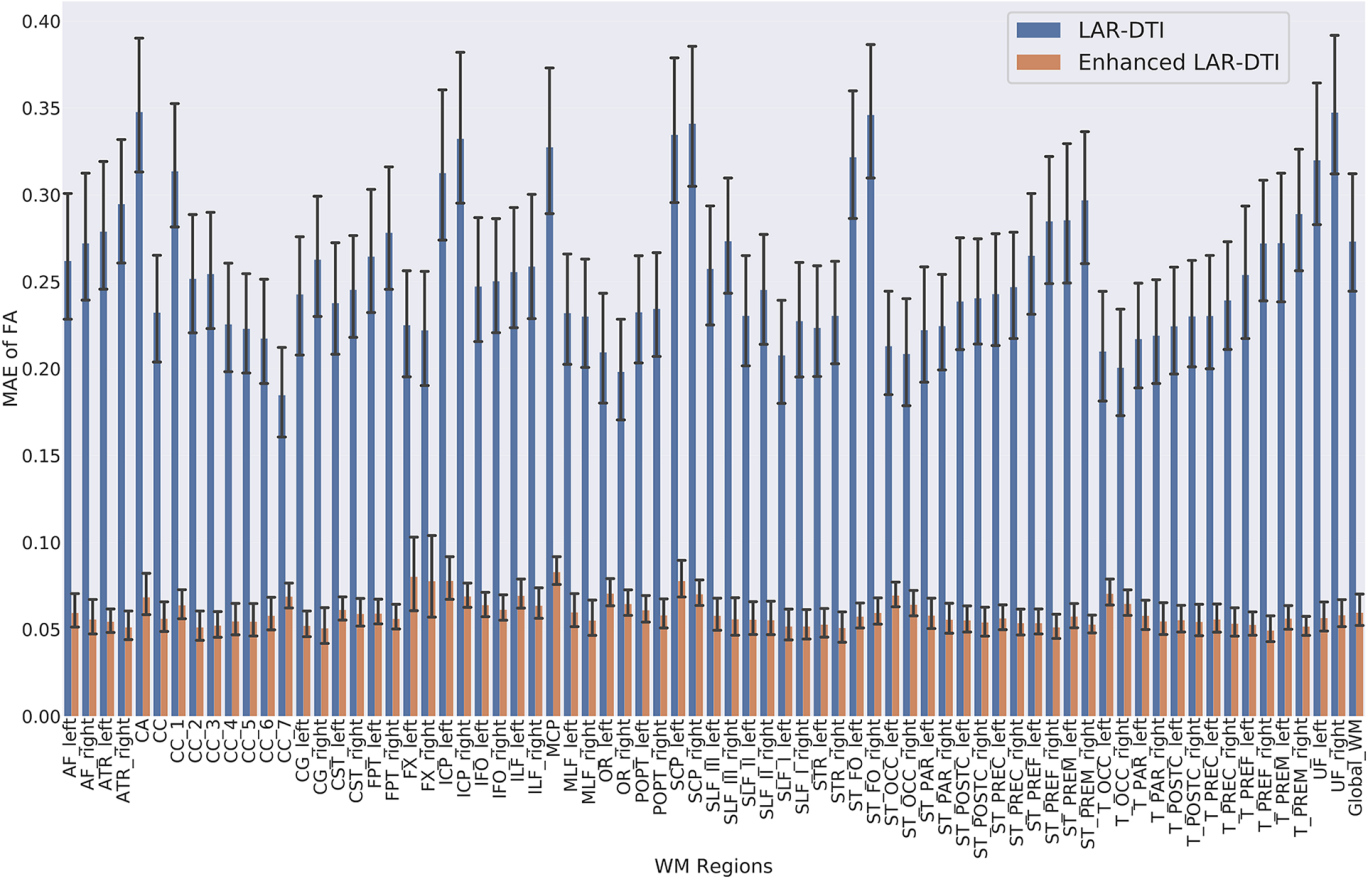
Mean absolute FA differences for LAR-DTI and enhanced LAR-DTI by HADTI-Net in 72 WM tracts and entire WM region. Analysed WM tracts in order of apperance: arcuate fascicle (AF), anterior thalamic radiation (ATR), commissure anterior (CA), corpus callosum (CC) and its subregions (rostrum, genu, rostral body, anterior midbody, posterior midbody, isthmus, and splenium), cingulum (CG), corticospinal tract (CST), Middle longitudinal fascicle (MLF), fronto-pontine tract (FPT), fornix (FX), inferior cerebellar peduncle (ICP), inferior occipito-frontal fascicle (IFO), inferior longitudinal fascicle (ILF), middle cerebellar peduncle (MCP), optic radiation (OR), parieto-occipital pontine (POPT), superior cerebellar peduncle (SCP), superior longitudinal fascicle (SLF), superior thalamic radiation (STR), uncinate fascicle (UF), thalamo-prefrontal (T_PREF), thalamo-premotor (T_PREM), thalamo-precentral (T_PREC), thalamo-postcentral (T_POSTC), thalamo-parietal (T_PAR), thalamo-occipital (T_OCC), striato-fronto-orbital (ST_FO), striato-prefrontal (ST_PREF), striato-premotor (ST_PREM), striato-precentral (ST_PREC), striato-postcentral (ST_POSTC), striato-parietal (ST_PAR), and striato-occipital (ST_OCC).

As discussed in the previous experiment, FA is highly sensitive to microstructural changes (31) and is significantly heavily impacted due to the low angular resolution of DWI. As demonstrated in the previous section, HADTI-Net’s prediction can mitigate the overall differences in the WM region. However, since microstructural changes may be regional depending on the neurological disorder, we would like to further explore the performance of HADTI-Net to reconstruct FA measures for relevant WM tracts. Specifically, the MAE of FA was calculated on each WM tract bundle provided by TractSeg when comparing LAR-DTI and enhanced LAR-DTI to HAR-DTI, as shown in Figure 4. The experimental results show that the enhanced LAR-DTI reduces the average MAE of FA in all the WM tract bundles to below 0.1, while LAR-DTI presented average MAE values ranging from 0.15 to 0.4. These results, further demonstrate that the improvements shown in Section 3.1 are not just a global phenomenon for the WM region, but rather all WM tracts present a more accurate representation of their anisotropy. This is encouraging news as some of these tracts overlap leading to regions with crossing fibres where the lack of gradient information could lead to erroneous FA estimates, even if the gradient directions are evenly distributed in the hemisphere. However, HADTI-Net is capable of reducing errors to a similar level for any WM tract.

### 3.3. Clinical impact

**Figure 5:**
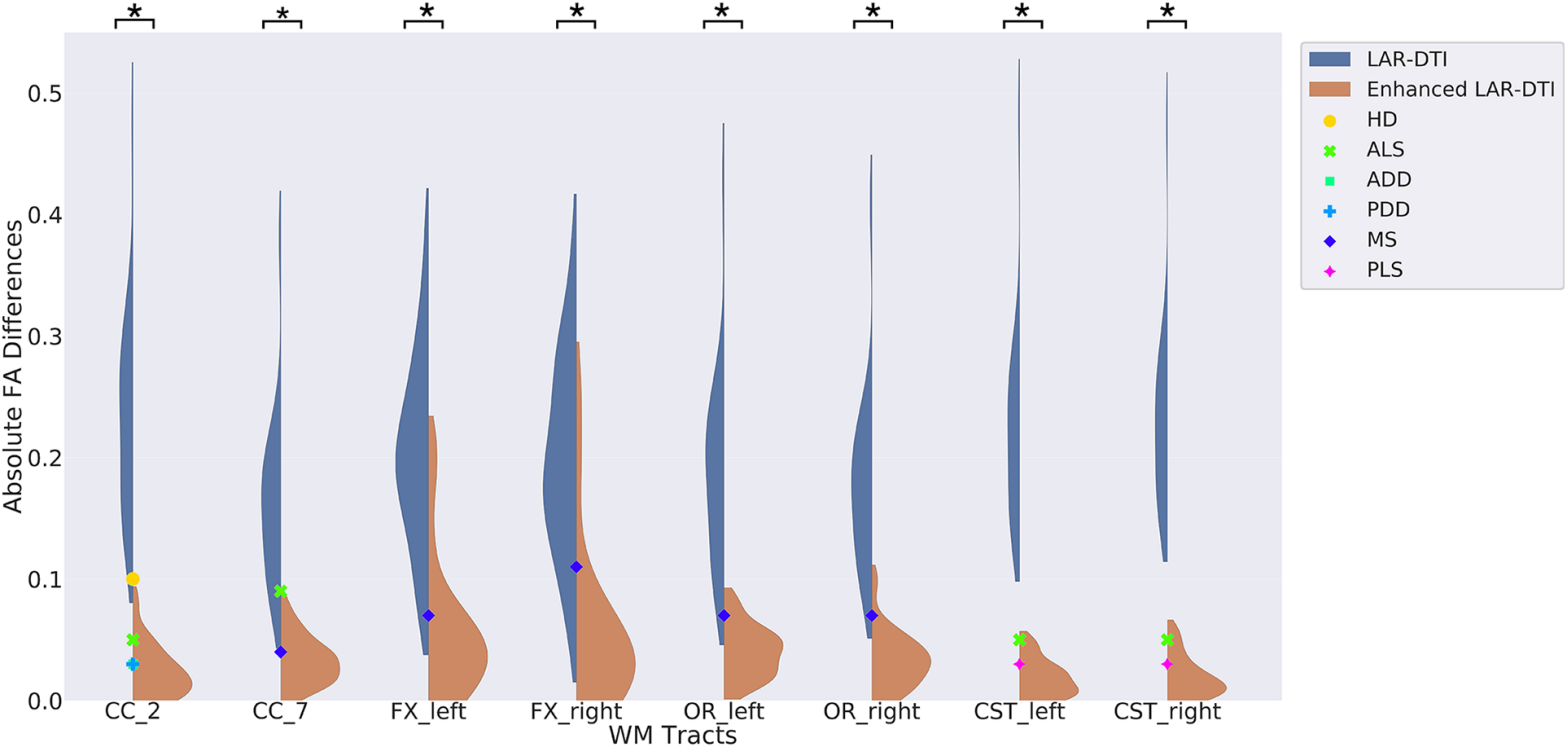
The distribution of the absolute FA differences when comparing LAR-DTI and enhanced LAR-DTI by HADTI-Net to HAR-DTI for all the testing subjects in each individual WM tract. The reported FA differences for different brain disorders are marked with corresponding annotations. Significant differences between LARDI and enhanced LAR-DTI values inside the selected WM tracts are marked on the top of each violin plot with a ∗ (p<0.01).

To underscore the potential clinical significance of our work, evidence was gathered from relevant works to carry out further analysis. In particular, different clinical studies have demonstrated that aging-related neurodegeneration can lead to FA reduction (32), and a severe cognitive impairment can cause a significant decrement of FA (33). Moreover, FA differences in specific WM tracts were observed in various brain diseases between age and gender-matched healthy controls and patients, including Huntington’s disease (HD), amyotrophic lateral sclerosis (ALS), Alzheimer’s disease dementia (ADD), Parkinson’s disease with dementia (PDD), multiple sclerosis (MS), and primary lateral sclerosis (PLS). For analysis purposes, these clinical differences were collected and reported with our experimental results in Figure 5, to compare the distribution of the observed absolute FA difference when comparing LAR-DTI and enhanced LAR-DTI to HAR-DTI for all the testing subjects in specific WM tracts related to corresponding neurological disorders. The goal of the experiment was to compare the distribution of the observed differences (errors), to the clinical differences observed in clinical studies (34). The hypothesis is that if the distribution of the differences between FA values between HAR-DTI and LAR-DTI (specifically, the mean) is higher than the clinically observed differences between subjects, the measurement errors would make it impossible to find these group-wise differences.

The figure demonstrates that LAR-DTI has a higher mean and a wider distribution of FA differences, which highlight the limitations of using a LARDI acquisition in clinical studies without further enhancement. For instance, a 0.1 FA difference was observed in the Genu tract (CC_2) between healthy controls and HD patients (35), where the FA differences for most LAR-DTI subjects are above 0.1. This could potentially impact the observed group differences between healthy controls and patients. Similar results can be concluded for the same tract in ALS and ADD patients (36, 37), the Splenium tract (CC_7) in ALS and MS patients (38), the Fornix tract (FX_left and FX_right) and the Optic radiation tract (OR_left and OR_right) in MS patients (39), and the Corticospinal tract (CST_left and CST_right) in ALS and PLS patients (40). The proposed enhancing deep learning network is capable of reducing measurement errors to a level lower than the gap between controls and patients, which suggests the ability to still measure these group-wise differences after the prediction of the enhanced LAR-DTI volume. Paired t-tests were further conducted between FA values of LAR-DTI and enhanced LAR-DTI for each reported WM tract that showed the ability of HADTI-Net to significantly reduce the FA errors brought by a LARDI acquisition (p<0.01, annotated as ∗ in the figure).

### 3.4. Generalisation test

**Figure 6:**
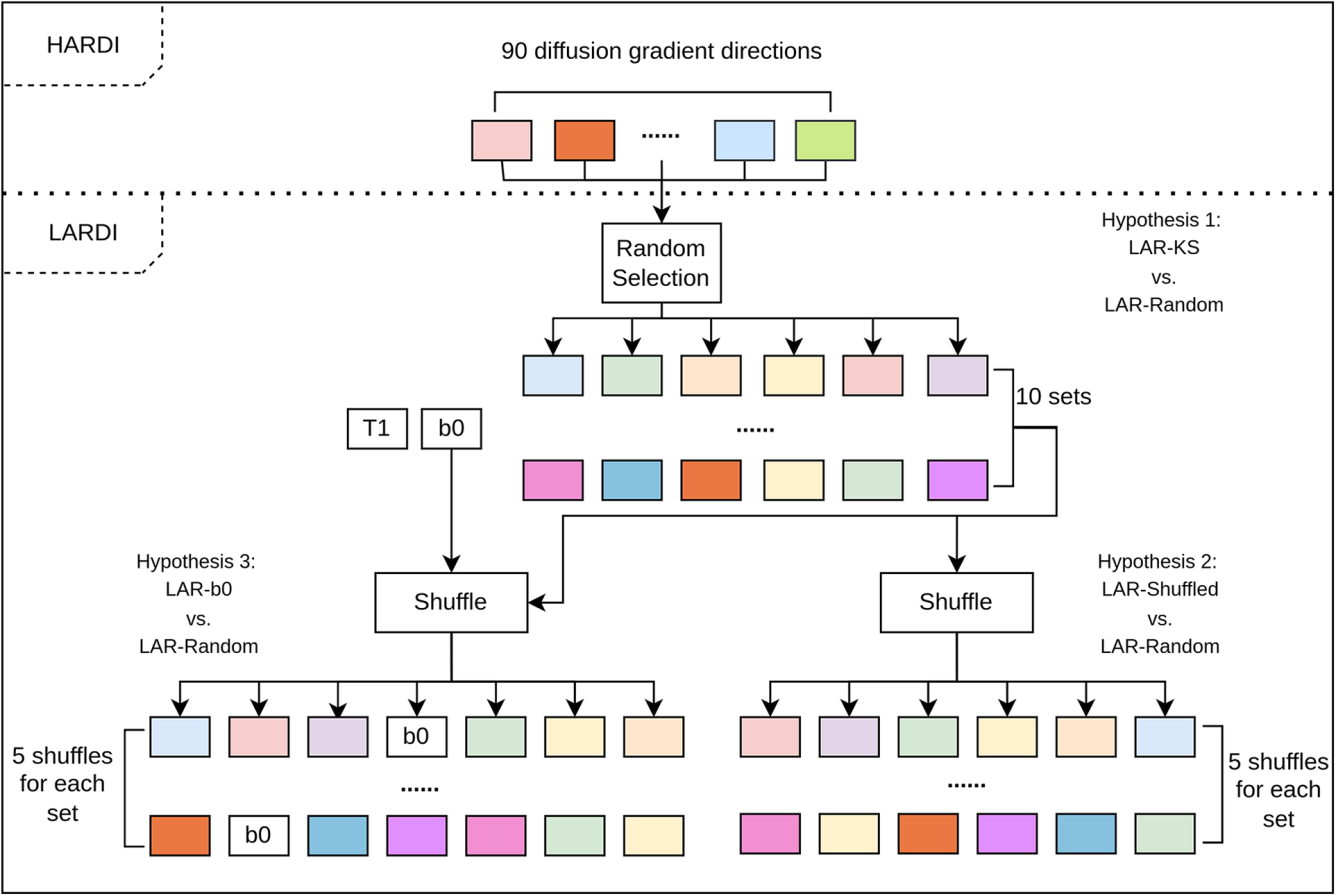
The flow chart for the genralisation experimental design and data preparation. Each set of inputs is then fed to the trained HADTI-Net to generate the enhanced LAR-DTI estimates and the MAE of FA when compared to HAR-DTI is calculated.

We have shown that HADTI-Net can estimate an improved DTI model from LARDI with a fixed set of 6 diffusion gradient directions using the deterministic KS algorithm (referred to as LAR-KS). To further validate the proposed HADTI-Net, we conducted a set of experiments to understand the importance of the 6 sampled gradient directions during inference and whether our random sampling training strategy led to a more general framework for any 6 evenly distributed gradient directions. Specifically, we defined 3 hypotheses to statistically test. Similarly to Section 3.2

design and data preparation for this section are shown in Figure 6, and the details of each experiment are discussed in the following subsections.

#### 3.4.1. Hypothesis 1: HADTI-Net is robust to the choice of gradient directions as long as they are evenly distributed

For this experiment, we define two sets of testing images. On the one hand, we have the LAR-KS images previously analysed in Sections 3.1–3.3. On the other hand, we have a new set of images with 6 randomly selected but evenly distributed gradient directions using our extension of the KS algorithm described in Section 2.1.3 (referred to as LAR-Random). Specifically, we randomly chose 10 different starting directions and sampled the other 5 directions using the KS algorithm. Both sets of images were then combined with T1 and b0 and passed into the HADTI-Net model and their FA MAE was calculated.

Since these sets of images cannot be paired (the FA of different random samplings are not measurements of the same property) and due to the lack of normalcy for the LAR-Random measurements we designed a subject-wise statistical test based on the Wilcoxon signed test. Specifically, for each subject, we designated the LAR-KS MAE as the “ground truth” estimate and subtracted it from the LAR-Random estimates. This gave us a distribution of the differences between LAR-KS and LAR-Random. Therefore, we could formulate our null hypothesis as the distribution of these values to be symmetrical around 0 (meaning that LAR-Random does not obtain lower or higher errors). After testing for each subject independently and correcting for multiple comparisons (20 subjects), no significant difference was found with a significance value after correction of α=0.0005, p-value∈(0.005,0.45)) and therefore we could not reject the hypothesis of systematic differences between the ideal evenly distributed sampling (LAR-KS) and a random set of evenly distributed directions (LAR-Random).

#### 3.4.2. Hypothesis 2: HADTI-Net is not sensitive to the order of the gradient directions in the input tensor

While the previous experiment proved that the sampling is not important, it did not necessarily prove that different orderings on the input vector would not lead to different results. While our modified KS algorithm introduces an element of randomness (by choosing the first gradient direction), the sampling is still deterministic for the other 5 gradient directions. Furthermore, the ordering of the directions is not relevant and the network should not try to learn that. To test this hypothesis, we created a new testing dataset called LAR-Shuffled, where we conducted 5 random shufflings for the 6 gradient directions in each DWI image within the 10 sets of LAR-Random, leading to 50 different sets of input images. Similarly to the previous experiment, the images were combined with the T1-weighted and b0 images and fed to HADTI-Net to obtain an enhanced DTI and the MAE of FA values was calculated.

Once again, samples could not be paired and for each subject we compared the distributions between the set of LAR-Random and LAR-Shuffle errors. In that case, we formulated our null hypothesis as both sets have the same distribution of error values. Due to the lack of normalcy for both sets, we performed a Mann-Whitney U test for each subject independently and corrected for multiple comparisons. Once again, we found no significant differences between the error values for any of the subjects (with a significance value after correction of α=0.0005, p-value∈(0.005,0.49)) meaning that we could not reject the null hypothesis of both distributions being equal.

#### 3.4.3. Hypothesis 3: the network depends on the b0 information and is able to locate the position of b0 in the input vector

While we have proven that the sampled directions are not important as long as they are evenly distributed (according to our variations of the KS algorithm), the question still remains of whether the network exploits the relationship between b0 and the diffusion-weighted information (which is a key part of the common algorithm to fit a DTI tensor). To test this hypothesis, we need to assume that positional information in the input is important (i.e. the order of the given channels). Unlike self-attention mechanisms or graph convolutions that do not rely on positional information in a sequence, convolutional and linear layers have different weights for different channels. We have proven with the previous experiment, that this is not the case, at least for the last 6 channels. However, that does not prove that the first two channels do not have specific weights (implying that the network knows the difference between diffusion-weighted images and structural ones). Therefore, assuming that the position of b0 is important, we now create a new set of files once again based on the LAR-Random set of images. Specifically, for each image in the LAR-Random set we randomly place b0 as one of the diffusion-weighted images (refer to as LAR-b0). T1 was then combined as the first image again and the input was fed to the network to estimate the enhanced DTI and calculate the FA MAE.

Similarly to the previous experiment, two sets of unpaired errors were obtained per subject. Therefore, we could reformulate our hypothesis as the distributions of errors for LAR-b0 and LAR-random being equal and perform a Mann-Whitney U test per subject (corrected for multiple comparisons). Unlike in the previous experiment, we found significant differences for all the subjects (with a significance value after correction of α=0.0005, $p\text{-value}\in (0,3.53\times 10^{-18})$), rejecting the hypothesis that the errors were equal. In fact, the results were significantly worse and close to the MAE for LAR-DTI from Table 1 suggesting that the network treats b0 and the diffusion-weighted images differently.

## 4. Conclusion

In this work, we have proposed HADTI-Net, a deep learning network to estimate the enhanced DTI model from LARDI with a set of minimal evenly distributed diffusion gradient directions. We conducted extensive experiments on subjects from the HCP dataset to validate the feasibility of applying HADTI-Net to generate enhanced DTI volumes and to evaluate the randomised training strategy for generalisation to any set of 6 directions. These results have shown comparable DTI scalar measures when compared to those from HARDI on the whole WM region and a large improvement for each WM tract independently. Furthermore, our quantitative analysis of WM tracts from a clinical perspective demonstrated that HADTI-Net has clinical significance by mitigating the FA errors brought by a set of minimal diffusion gradient directions. Finally, a set of statistical tests have proven that the network could generalise to any set of evenly distributed directions while highlighting the ability to distinguish between b0 and diffusion-weighted information. In conclusion, HADTI-Net can be used as a post-scanning technique to allow clinical studies with a set of minimal evenly distributed diffusion gradient directions while achieving reliable DTI metrics, comparable to a HARDI acquisition.

## Data Availability

The original contributions presented in the study are included in the article/supplementary material, further inquiries can be directed to the corresponding author/s. All the datasets used in the experiments are from Human Connectome Project (HCP), which can be accessed from https://www.humanconnectome.org/.
